# Predictors of loss to follow-up among patients on ART at a rural hospital in KwaZulu-Natal, South Africa

**DOI:** 10.1371/journal.pone.0177168

**Published:** 2017-05-24

**Authors:** Rachel Arnesen, Anthony P. Moll, Sheela V. Shenoi

**Affiliations:** 1 Jackson Institute for Global Affairs, Yale University, New Haven, Connecticut, United States of America; 2 Antiretroviral Programme, Church of Scotland Hospital, Tugela Ferry, KwaZulu-Natal, South Africa; 3 Section of Infectious Diseases, AIDS Program, Yale University School of Medicine, New Haven, Connecticut, United States of America; Universita degli Studi di Roma Tor Vergata, ITALY

## Abstract

**Introduction:**

Improved HIV outcomes as a result of expanded antiretroviral therapy (ART) access is threatened by increasing rates of loss to follow up (LTFU) among those on ART, largely reported in urban populations. Some reports suggest that LTFU rates are overestimated due to patient movement to other facilities and inadequate medical records.

**Study objective:**

To define the proportion disengaging from HIV care as well as the characteristics of those LTFU in order to design and implement appropriate interventions to increase retention.

**Methods:**

We performed a retrospective review of patients who discontinued ART at a central hospital ART clinic in rural South Africa and compared with patients receiving care at the 15 primary health clinics (PHCs) to determine the true proportion of those who were LTFU. We also compared those who discontinued ART with those who did not at the central hospital ART clinic to determine predictors of loss to follow up.

**Results:**

Among 3242 patients on ART, 820 were originally marked as LTFU. Among all patients, 272 (8.4%) were found at a clinic on treatment, 56 (1.7%) were found at a clinic from which they had since discontinued treatment, and 10 (0.3%) returned to care between June and July 2016, leaving 475 (14.7%) unaccounted for and thus categorized as ‘true’ LTFU. Factors found to be associated with discontinuation include being male, age 18–35, having a CD4 count under 200 cells/μL, and being on ART for under six months.

**Conclusions:**

Young men with low CD4 counts early after ART initiation are at highest risk of ART disengagement in this rural South African HIV clinic. Novel interventions targeting this group are needed to improve retention in care.

## Background

Substantial progress has been made globally in the fight against HIV. The number of new infections has decreased from a peak of 2.7 million per year in 2005 to 2.0 million per year in 2014 11], accompanied by an increase in access to antiretroviral therapy (ART). In 2000, only 700,000 people were on ART globally compared to 15.8 million in mid-2015 [1]. Approximately 7 million people are currently living with HIV in South Africa, with a 19.2% prevalence rate among adults age 15–49 [1]. In 2014, 180,000 South African deaths were attributable to AIDS [1]. ART access has increased dramatically over the past 15 years in South Africa, from only 4,000 patients on treatment in 2000 to over 3.1 million on treatment in 2014 [1].

Surveillance studies in South Africa estimate that 62.2% of male and 45% of female persons living with HIV are unaware of their HIV+ status [2]. Until recently, HIV+ individuals were only eligible for ART in South Africa if they met CD4 count criteria. Under previous criteria restricting ART to those with CD4<350, 59% of those eligible were not receiving ART [2]. Since September 2016, South Africa has progressively implemented a test-and-treat policy in which every individual with a positive HIV test result would be eligible to receive ART immediately, regardless of CD4 count. Thus, strategies are needed to ensure that more individuals are aware of their HIV status and are linked to and retained in care. Currently, approximately 65–80% of patients are retained in HIV care in resource-limited settings (RLS) [3, 4, 5, 6]. Factors associated with loss to follow-up among HIV+ patients on ART include dissatisfaction with health services, lack of financial resources to access treatment, stigma, improving health condition, male gender, baseline CD4 count greater than 200 cells/ml, follow-up CD4 count less than 200 cells/ml, and confidentiality concerns [7, 8, 9, 10, 11, 12, 13].

Rates of loss to follow up among those on ART are approximately 10–20% [14, 15, 16]. However, some reports suggest that loss to follow up rates are overestimated due to patient movement to other facilities and inadequate medical records [14, 17, 18]. Without more precise data about the rate of LTFU as well as the characteristics of those who disengage from treatment, appropriate interventions to increase ART adherence cannot be designed and implemented. While previous studies have investigated characteristics of those who are LTFU in South Africa, questions still remain about the dynamics of LTFU in rural settings. Accordingly, we sought to more accurately characterize movement between a central hospital ART clinic and its associated primary health clinics (PHCs) in a rural region of South Africa to determine the proportion and characteristics of patients who were no longer on ART and therefore considered “true” loss to follow up.

## Methods

### Setting

The study was conducted at the Church of Scotland Hospital (COSH) HIV clinic in Tugela Ferry, South Africa. COSH is a 350-bed district hospital which serves Msinga sub-district of KwaZulu-Natal, South Africa and receives referrals from 15 PHCs. Msinga has a population of approximately 180,000 and is characterized by extreme poverty. Unemployment rates are approximately 85%, 70% of people have no access to electricity, and 70% have no access to clean water [19]. At the time of this study, adults were considered eligible for ART following HIV+ diagnosis with CD4 count of <500 cells/μL. At COSH, patients are typically given 1-month ART prescriptions in which they have to physically come to the clinic each month to receive their medication refills [20]. However, once stable on ART for 12 months, patients can be enrolled in Medipost, signifying monthly refills from designated ART dispensing stations and clinic visits once every six months [20, 21].

### Study design

We performed a retrospective review of patients who discontinued ART at the central hospital HIV clinic and compared with patients receiving care at the 15 PHCs to determine the true proportion of LTFU. We also compared those who discontinued ART with those who did not at the central hospital clinic to determine characteristics associated with LTFU.

### Data collection and measures

Data on patients initiating HIV care at COSH is recorded in an electronic database called tier.net [22]. Doctors and nurses record patient information at each visit into a paper file which is then entered into the computer system by a data capturer. Each of the 15 PHCs has a tier.net database, but they are not linked to each other or to COSH. We reviewed the tier.net database from COSH and generated a list of patients who discontinued ART from January 2015-June 2016, defined as patients who had not collected ART for a minimum of 90 days.

Patients located at the PHCs were categorized as either “found on treatment,” defined as actively receiving treatment at that clinic or having transferred out while actively taking the treatment, or as “found not on treatment,” defined as patients who were listed in the PHC database but who had since become LTFU from that clinic. Outcomes were censored as of July 1, 2016. A comparison list of those who were retained in care was generated from COSH’s tier.net database. Patients retained in care were defined as patients who were on ART and collecting medication without LTFU for at least three months from January 2015 to June 2016. Abstracted data included age, gender, months on ART, and most recent CD4 count and viral load, for both those who were LTFU and those who were retained in care. Double data entry was used to ensure data quality and patients categorized as lost to follow up were confirmed with staff at each clinic.

### Data analysis

Chi-square and Kruskal-Wallis analyses were performed to determine predictors of LTFU. Using a threshold of p<0.1, we conducted a multivariable regression analysis to determine independent predictors of LTFU. All analyses were performed using SAS Version 9.4.

The study was approved by the South African Medical Association Research Ethics Committee (#1401013255) and Yale University Human Investigations Committee (#1401013255). The data reviewed and presented in this study was originally collected in medical records as part of routine clinical care. Informed consent was not requested of patients since recording of data into the medical charts was for the purpose of regular medical care.

## Results

### Sample characteristics

**Fig 1:**
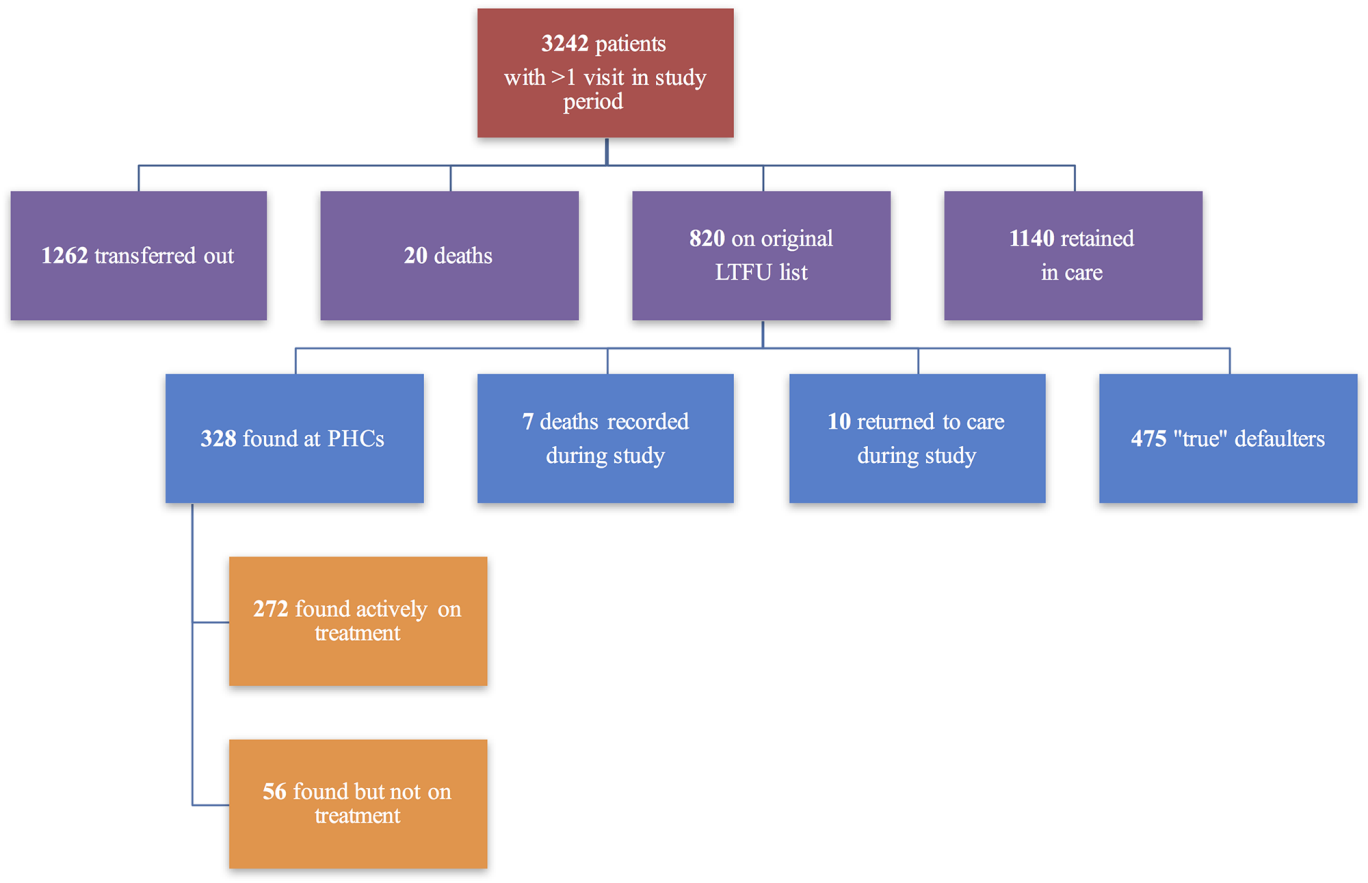
Patients with ≥1 Visit to COSH HIV clinic from January 2015-June 2016.

Of all 3242 patients who visited the COSH central hospital HIV clinic at least once from January 2015 to June 2016 (Table 1), the majority were female (57.6%) and between 18–35 years old (33.4%). Among all patients with at least one visit, 1140 (35.1%) remained on treatment, 1262 (38.9%) transferred out to another clinic, 20 (0.6%) died, and 820 (25.2%) were recorded as LTFU (Fig 1).

**Table 1:** Characteristics of all HIV infected patients on ART at the hospital HIV clinic from January 2015-June 2016.

n = 3242	n	(%)
**Outcome**		
Retained in care	1140	(35.2%)
Transferred out	1262	(38.9%)
Died	27	(0.8%)
Lost to follow-up	475	(14.7%)
Found at clinics on treatment	272	(8.4%)
Found at clinic not on treatment	56	(1.7%)
Returned to care during study	10	(0.3%)
**Sex**		
Male	1374	(42.4%)
Female	1868	(57.6%)
**Age**		
<18	526	(16.2%)
18–35	1082	(33.4%)
35–45	962	(29.7%)
≥45	668	(20.6%)
Unknown	4	(0.1%)
**Most Recent CD4 Count**		
<200	636	(19.6%)
200–500	859	(26.5%)
≥500	761	(23.5%)
Unknown	986	(30.4%)
**Most Recent Viral Load**		
<200	984	(30.4%)
200–1000	277	(8.5%)
≥1000	397	(12.2%)
Unknown	1584	(48.9%)
**Months on ART**		
<6	129	(4.0%)
6–11	269	(8.3%)
12–23	504	(15.5%)
≥24	1951	(60.2%)
Unknown	389	(12.0%)

### Finding LTFU at primary health clinics

Among the 820 individuals originally marked as LTFU, 272 (8.4%) were found at a clinic on treatment, 56 (1.7%) were found at a clinic from which they had since become LTFU, and 10 (0.3%) returned to care between June and July 2016, leaving 475 (14.7%) unaccounted for and thus categorized as ‘true’ LTFU (Fig 1).

With regards to those LTFU (n = 475), the majority (51.4%) were male (Table 2). A majority of those LTFU were between the ages of 18 to 45 (70.3%). This group of patients who became LTFU had a median time on ART of 20 months (IQR 10–42). The clinics at which patients were located varied in the number of patients found and the ratio of patients found on treatment to found not on treatment. The highest number of patients were located at Gateway (73), Pomeroy (50), and Mhlangana (31) clinics (Table 3). Only 75% of the patients found at Gateway were still on treatment, compared to 82% at Pomeroy and 97% at Mhlangana, suggesting potential differences in retention in care at the clinic level.

**Table 2:** Characteristics of patients who were LTFU at the hospital HIV clinic from January 2015-June 2016.

n = 475	n	(%)
**Sex**		
Male	244	(51.4%)
Female	231	(48.6%)
**Age**		
<18	69	(14.5%)
18–35	188	(39.6%)
35–45	146	(30.7%)
≥45	72	(15.2%)
**Most Recent CD4 Count**		
<200	142	(30.0%)
200–500	118	(24.8%)
≥500	76	(16.0%)
Unknown	139	(29.3%)
**Most Recent Viral Load**		
<200	89	(18.7%)
200–1000	28	(5.9%)
≥1000	48	(10.1%)
Unknown	310	(65.3%)
**Months on ART**		
<6	62	(13.1%)
6–11	67	(14.1%)
12–23	105	(22.1%)
≥24	190	(40.0%)
Unknown	51	(10.7%)

**Table 3:** Number and proportion of patients located at primary care clinics.

	Found on treatment (n = 272)	Found not on treatment (n = 56)
Collessie	7 (2.6%)	7 (12.5%)
Cwaka	15 (5.5%)	0
Ethembeni	29 (10.7%)	3 (5.4%)
Gateway	55 (20.2%)	18 (32.1%)
Gunjana	19 (7.0%)	3 (5.4%)
Mandleni	11 (4.0%)	4 (7.1%)
Mawele	2 (0.7%)	1 (1.8%)
Mazabeku	6 (2.2%)	0
Mbangweni	22 (8.1%)	2 (3.6%)
Mhlangana	30 (11.0%)	1 (1.8%)
Mumbe	7 (2.6%)	3 (5.4%)
Ngubevu	14 (5.1%)	2 (3.6%)
Nocomboshe	7 (2.6%)	2 (3.6%)
Pomeroy	41 (15.0%)	9 (16.1%)
Qinelani	7 (2.6%)	1 (1.8%)

### Comparing patients who became lost to follow-up and those retained in care

In total, we evaluated and compared 475 patients who became LTFU and 1140 who were retained in care (Table 4). Males accounted for 51% of LTFU compared to 40% of those retained in care (p = 0.001), the median number of months on ART for those LTFU was 20 (IQR 10–42) compared to 48 for those retained in care (IQR 26–70, p<0.0001), the most recent CD4 count for those LTFU was 248 (IQR 94–436) compared to 449 (IQR 260–693, p<0.0001) for those retained in care. The percentage of those LTFU virally suppressed was 54% compared to 64% of those retained in care (p = 0.01). There was a trend towards significance with regards to age, with a median age of those LTFU of 34 years (26.7–41) and of those retained in care 35.7 years (IQR 24.9–44, p = 0.08).

**Table 4:** Characteristics of patients on ART lost to follow-up and patients retained in care at the hospital HIV clinic from January 2015 –June 2016.

Characteristics	n	LTFU (n = 475)	Retained in care (n = 1140)	p-value
Age, median years (IQR)	1615	34.0 (26.7–41.0)	35.7 (24.9–44.0)	0.08
<18	289	8.9 (2.5–14.5)	10.5 (6.8–14.2)	0.05
18–35	519	30.4 (26.4–32.6)	29.7 (25.4–32.1)	0.67
35–45	483	39.2 (37.4–42.1)	40.3 (37.8–42.4)	0.08
>45	324	52.5 (48.1–59.3)	51.2 (47.8–56.5)	0.29
Male gender (n, %)	1615	244 (51%)	685 (40%)	0.001
Months on ART total (median, IQR)	1614	20 (10–42)	48 (26–70)	<.0001
Most recent CD4 count, cells/uL (median, IQR)	1318	248 (94–436)	449 (260.3–693)	<.0001
Last VL <200 (n, %)	1101	89 (54%)	601 (64%)	0.01

### Lack of laboratory monitoring

Only 62.2% of patients LTFU and retained in care had complete data on all variables of interest. While 81.6% of individuals either LTFU or retained in care (n = 1615, Table 4) had at least one CD4 measurement recorded in tier.net (86.1% of those retained in care, 70.7% of patients LTFU), only 68.2% of those LTFU and retained in care had a VL measurement recorded (82.1% of those retained in care, 34.7% of those LTFU). Among those became LTFU, the median time from last CD4 measurement to LTFU was 8 months (IQR 4–18). Among those retained in care, the median time from last CD4 measurement to last visit was 6 months (IQR 1–18). The number and proportion of all individuals seen at COSH (n = 3242), including patients who officially transferred out of care, with missing VL and CD4 data can be seen in Table 1.

### Predictors of LTFU

Upon univariate analysis, we found that ages 18–45, male gender, CD4 <500, and ART <24 months were significantly associated with ART disengagement (Table 5). Males were 1.6 (95% CI 1.3–2.0) times more likely to become LTFU than females. Those in the age 18 to 35 group were 2.0 (1.5–2.7) times more likely to disengage compared to those over age 45. Patients with a most recent CD4 count of under 200 were 4.3 (3.1–6.0) times more likely to become LTFU than those with a CD4 count of above 500. Those with a most recent viral load of above 1000 were 1.5 (1.0–2.3) times more likely to disengage than those with a most recent viral load of less than 200. Patients on ART for less than six months had a 10.3 (6.4–16.5) times higher likelihood of disengagement.

**Table 5:** Predictors of LTFU.

	Unadjusted OR (95% CI)	Adjusted OR (95% CI)
Sex		
Male	1.59 (1.28–1.97)*	1.31 (.91–1.89)
Female	Reference	Reference
Age		
<18	1.10 (.75–1.60)	1.65 (.89–3.07)
18–35	2.00 (1.45–2.73)*	1.79 (1.05–3.07)*
35–45	1.52 (1.09–2.10)*	1.57 (.92–2.67)
≥45	Reference	Reference
CD4 Count		
<200	4.34 (3.12–6.03)*	2.64 (1.61–4.35)*
200–500	1.69 (1.23–2.33)*	1.24 (.80–1.88)
≥500	Reference	Reference
Viral Load		
<200	Reference	Reference
200–1000	1.54 (.96–2.45)	1.37 (.84–2.24)
≥1000	1.53 (1.04–2.25)*	.99 (.63–1.55)
Months on ART		
<6	10.30 (6.42–16.53)*	2.83 (1.02–7.82)*
6–11	3.06 (2.16–4.32)*	.45 (.20–1.02)
12–23	3.88 (2.87–5.25)*	1.35 (.80–2.25)
≥24	Reference	Reference

*significant at p≤0.05

However, on multivariate analyses, the only factors that remained independent predictors of ART disengagement were age 18–35, CD4 count under 200, and ART for less than 6 months (Table 5). Those aged 18–35 were at a 1.8 (1.1–3.1) times higher risk of disengaging compared to those over age 45. Patients with a CD4 count below 200 were at a 2.6 (1.6–4.4) higher risk of LTFU compared to those with a CD4 count of over 500, and those on ART for under 6 months had a 2.8 (1.0–7.8) higher risk of LTFU compared to those on ART for over 2 years.

## Discussion

This study characterized disengagement from a central hospital ART clinic in rural KwaZulu-Natal, South Africa. The overall “true” LTFU rate from the central hospital HIV clinic was 14.7% within the Msinga sub-district. Significant differences between those who became LTFU and those who were retained in care exist with respect to gender, time on ART, most recent CD4 count, and proportion virally suppressed (defined as having a VL of under 200). Independent correlates of disengagement include male gender, age 18–35, lower CD4 count (<200) and shorter duration on ART (<6 months). While male gender was only borderline significant, the higher risk for younger males may partially be explained by the mobile nature of the population in Tugela Ferry. Employment opportunities are limited in this rural impoverished region and thus males of working age frequently travel long distances to work in other areas such as Johannesburg for months at a time. If these patients did not request a formal transfer of care, they would be recorded as LTFU. Though some of these patients likely attended another clinic and continued ART, others simply stopped ART while away from home. [23, 24]

Additionally, patients with more advanced HIV disease were most likely to disengage. In particular, those with CD4 counts of below 200 cells/mm^3^ were at highest risk, nearly three times more likely to stop ART. Previous studies have found mixed results on the impact of CD4 count, with evidence for both higher and lower CD4 counts increasing the risk of LTFU [8, 9, 11, 12]. There are multiple possible explanations for our findings. First, patients with CD4 counts of below 200 cells/mm^3^ may have died within three months of their last clinic visit, an outcome we were unable to confirm in this study. A previous South African study found that among 23% of patients who were LTFU, 48.8% had died [8], though was unable to ascertain date of death and thus could not determine whether death occurred within three months of their last clinic visit. A meta-analysis of ART programs found a combined mortality rate of 46% among patients lost to follow-up, though there were significant differences between studies [25]. In our study as well, dates of death were not available, so the impact of mortality is unknown. However, these findings suggest that the significant association of low CD4 count with disengagement may at least partially be attributed to unreported patient death and not LTFU.

Another possible explanation is that patients with low CD4 counts who are on ART may not be taking their medication as prescribed. Lack of clinical response to treatment may reinforce poor adherence and result in stopping ART altogether. Patients may also have baseline resistance to first line ART or develop resistance to ART, further decreasing the effectiveness of therapy and resulting in poor clinical response and bolstering patient notions of lack of ART effectiveness. Furthermore, individuals on ART for less than six months were also significantly more likely to disengage than those on treatment for longer lengths of time. This high initial attrition rate, demonstrated in other studies [3, 26], indicates that more targeted interventions early after ART initiation may be necessary to assist patients on treatment through the first six months. Moreover, though not measured in this retrospective record review, stigma remains a powerful contributor to lost to follow up, especially where patients may be viewed receiving treatment in their community [27]. In addition to the factors discussed above, this may explain the findings that patients disengaged from HIV care after transferring to their local PHC. Future studies, particularly among those in RLS who are LTFU soon after ART initiation, should examine reasons for treatment discontinuation [10, 28, 29, 30, 31] to inform targeted adherence interventions that are feasible [32, 33, 34, 35] in these settings.

Overall, a substantial proportion of patients on ART lacked a documented follow up CD4 and VL. South African guidelines stipulate that all HIV+ patients should have a CD4 count done upon ART initiation and every six months subsequently, and have a VL test done every six months post-ART initiation. However, in our rural South African primary health clinics, documentation was lacking and disproportionately affected those who became LTFU. It is unclear from this retrospective study whether this reflects inadequate systems to perform the test or record test results, or whether this is truly a marker for LTFU [36, 37, 38]. If the latter, lack of follow up testing could serve to identify patients at high risk of disengagement who need intervention.

Our data also suggest that there may be differences in retention according to primary care clinic. We speculate that clinic workload may play a role in differences in retention in care, though this data was not available in our review. These potential differences need to be evaluated and confirmed in future studies and the reasons for the disparities in retention among primary care clinics need to be explored.

We recognize several limitations to this study. First, national death registries were not able to be checked due to the lack of national ID numbers on many patient files. It is likely some patients on the true LTFU list have died. Secondly, we were only able to cross-reference the COSH LTFU list with the PHCs within the sub-district. We cannot account for patients who may have moved to other regions outside of Msinga. Third, our review focused on data recorded in the tier.net database, which unlike paper records, had a limited number of variables recorded. Data on clinical status of individual patients was not available. Fourth, the database recorded only the most recent CD4 count, precluding measuring impact of the nadir or baseline CD4 count. Furthermore, most patients are initiated on ART by nurses at primary care clinics in Msinga sub-district; the central hospital HIV clinic initiates patients who may have been recently admitted to the hospital or are otherwise more complicated and referred by the primary care clinics. Therefore, the study population may be different than the general population of ART initiators in this region and the results may not be generalizable. Further comparisons with those discontinuing ART at the primary care clinics are needed. Additionally, despite thorough efforts to account for duplication, it is possible that some duplicate files remained in the dataset. Lastly, this is a retrospective review of routine medical records and subject to missing data.

## Conclusion

Though we identified a slightly lower LTFU rate than in other studies, this study confirmed that retention in care is emerging as a major problem in under-resourced settings, similar to developed nations. Increased efforts to target those at risk, in particular men, those age 18–35, those having lower CD4 count (<200), and those on ART for a shorter period of time (<6 months), are needed to prevent LTFU and improve overall VL suppression, reduce transmission, and decrease HIV incidence. Increased emphasis is also needed on ensuring the performance and recording of indicated lab tests and improving linkage to death registry data. Future research that develops and evaluates strategies for reducing disengagement, particularly in the first six months after ART initiation, improving medication adherence, and reengaging those who have discontinued ART is warranted.

## Supporting information

S1 Text ART LTFU study data file. (XLSX)
